# ILC3: a case of conflicted identity

**DOI:** 10.3389/fimmu.2023.1271699

**Published:** 2023-10-17

**Authors:** Ivan Koprivica, Suzana Stanisavljević, Dragica Mićanović, Bojan Jevtić, Ivana Stojanović, Đorđe Miljković

**Affiliations:** Department of Immunology, Institute for Biological Research “Siniša Stanković” - National Institute of Republic of Serbia, University of Belgrade, Belgrade, Serbia

**Keywords:** innate lymphoid cells, phenotype, flow cytometry, humans, mice

## Abstract

Innate lymphoid cells type 3 (ILC3s) are the first line sentinels at the mucous tissues, where they contribute to the homeostatic immune response in a major way. Also, they have been increasingly appreciated as important modulators of chronic inflammatory and autoimmune responses, both locally and systemically. The proper identification of ILC3 is of utmost importance for meaningful studies on their role in immunity. Flow cytometry is the method of choice for the detection and characterization of ILC3. However, the analysis of ILC3-related papers shows inconsistency in ILC3 phenotypic definition, as different inclusion and exclusion markers are used for their identification. Here, we present these discrepancies in the phenotypic characterization of human and mouse ILC3s. We discuss the pros and cons of using various markers for ILC3 identification. Furthermore, we consider the possibilities for the efficient isolation and propagation of ILC3 from different organs and tissues for *in-vitro* and *in-vivo* studies. This paper calls upon uniformity in ILC3 definition, isolation, and propagation for the increased possibility of confluent interpretation of ILC3’s role in immunity.

## 1 Introduction

Group 3 innate lymphoid cells (ILC3s) have been identified as critical regulators of intestinal homeostasis and immune responses to both commensal and pathogenic bacteria in the gut. Subsequent studies showed that ILC3s are present in a variety of tissues and organs, including the lungs, skin, and lymphoid tissues. ILC3s are now recognized as important players in the immune response against different pathogens and are suggested to interfere with the development of inflammatory and autoimmune diseases (1, 2). The discovery of ILC3 has opened new avenues for the study of the role of the innate immune system in health and disease. Overall, the discovery of these cells represents a significant milestone in our understanding of the innate immune system.

ILCs were first identified in the early 2010s by researchers studying the immune system in the intestinal mucosa. Before being classified as a distinct cell population, they had been identified in mice and humans as cells that lack antigen-specific T-cell or B-cell receptors (TCR or BCR), expressing natural killer (NK) cell markers and producing various cytokines in response to stimulation. There are three populations of ILCs, namely, ILC1, ILC2, and ILC3, broadly corresponding to Th1, Th2, and Th17/Treg cells, respectively. Initially, ILC3s were described as RORγt-dependent NKp46^+^ NK cells that produce IL-22 and provide mucosal immune defense in the intestine of mice (3–5). At the same time, IL-2-producing NKp44^+^ NK cells were detected in human mucosa-associated lymphoid tissues, such as tonsils and Peyer’s patches (6). Due to the almost simultaneous discovery by several research groups, IL-22-producing natural cytotoxicity receptor-expressing (NCR^+^) cells have been referred to as NK22 (6, 7), NCR22 (8), NKR-Lti (9), or ILC22 (10). Given the similarities between IL-22-producing NCR^+^ ILC and NK cells, researchers sought to elucidate their developmental pathways. Although RORγt^+^ ILCs were originally thought to be a subpopulation of NK cells, cell fate-mapping studies showed that their developmental pathways were distinct. This conclusion is supported by the fact that NK cells do not express RORγt during development (8, 9). Further distinctions between ILC3 and NK cells are observed at the mature stage. Like T helper (Th) 17 cells, ILC3s express RORγt and aryl hydrocarbon receptor (AhR) and produce a high amount of IL-22 in response to IL-23. On the other hand, NK cells from peripheral or cord blood cultured under Th17 conditions with or without AhR ligands are unable to produce IL-22 (6). Additionally, under Th17-polarizing conditions, IL-22-producing NCR^+^ ILC cells do not produce IL-17 (6). In light of this, it has been suggested that ILC3s and NK cells undergo distinct differentiation pathways.

Since ILC3s express RORγt, another explanation was that they may develop from lymphoid tissue inducer (LTi) cells, previously known to also express RORγt (9). LTi cells are involved in the formation of lymphoid tissue during fetal development in mice and humans. While LTi cells express IL-17 and IL-22, NKp46^+^ ILCs express only IL-22 (11, 12). Moreover, LTi cells are distinguished from (NKp46^+/−^) ILCs by expressing markers such as CD4, CD25, and CCR6 on their surface (13, 14). In contrast to LTi, which are only found during fetal development, “adult LTi,” called LTi-like cells, can be found in the lamina propria of the intestine and in the tonsils, and they arise from bone marrow precursors (15). Although LTi-like cells and LTi are very similar in their expression profile, only the former express OX40L and CD30L (16). Evidence that NCR^+^ ILCs are a distinct lineage from LTi was provided by studies showing that RORγt^+^α4β7^+^ cells develop into CD4^+^ and CD4^−^ LTi, whereas RORγt^+^α4β7^−^ give rise to IL-22-producing NKp46^+^ cells in mice (12). Therefore, the term ILC3 was introduced by Spits and colleagues to describe the group 3 ILCs which are distinct from LTi (17). However, it is still not clear if NCR^+^ ILC3s have a different developmental pathway from LTi. It is generally presumed that LTi cells arise in the fetal liver and are primarily involved in developing lymphoid tissues, while NCR^+^ ILC3s develop from bone marrow lymphoid progenitors in the gut and lymphoid tissue. Accordingly, RORγt^+^CD34^+^α4β7^+^ hematopoietic progenitor cells have been defined in humans as IL-22^+^ ILC3-specific precursors (18). Still, there was a report that LTi cells gave rise to ILC3 in fetal lymph nodes (11), thus suggesting that the development of LTi and ILC3 is not strictly divergent. Furthermore, NCR^+^ ILC3 can be generated from RORγt^+^ precursors stemming from LTi-like cells. Indeed, in a study on mice, it was shown that NKp46^+^RORγt^+^ cells differentiate directly from LTi-like cells (NKp46^−^RORγt^+^) (9). Along the same line, it was demonstrated that human RORγt^+^NKp46^+^ cells can be generated *in vitro* from LTi-like cells obtained from adult tonsils (19).

As in mice, the development of ILC in humans begins with the common lymphoid progenitor (CLP), characterized as lineage(Lin)^−^CD34^+^CD45RA^+^CD10^+^KIT^–^, from which all lymphocyte subsets, including ILC, T, and B cells can develop. Intermediate stages of NK development are also thought to differentiate from this progenitor, at least in secondary lymphoid tissues, such as tonsils and lymph nodes (20). ILC differentiation further leads to the early tonsil progenitor cells (EToP), with two distinct phenotypes, EToP1 (Lin^−^CD34^+^CD10^+^KIT^−^) or EToP2 (Lin^−^CD34^+^CD10^+^KIT^+^) (21, 22). EToP2 can be divided into two groups based on ST2 (IL-33R, IL1r1) expression and on their ability to form ILC (21). Unlike ST2^−^ EToP, which can give rise to T cells and dendritic cells (DCs) *in vitro*, ST2^+^ EToPs give rise exclusively to ILC, both *in vitro* and *in vivo*, when isolated from pediatric tonsils and injected into immunodeficient NGS mice (22), and cannot give rise to non-ILC cells. Thus, these cells are the earliest human common ILC progenitors identified to date (22). Two other restricted progenitors were identified, one of which is a Lin^–^CD34^–^CD7^+^IL-7R^+^KIT^+^ ILC progenitor (ILCP), found in cord blood, fetal liver, peripheral blood, and other human tissues (23). This progenitor is common for ILC3, LTi, and ILC2 cells. Another progenitor, identified downstream of the ILCP, is CD34^–^KIT^+^CD56^+^, termed restricted ILCP (rILCP) (24), and can give rise to NK cells, ILC1, and ILC3, but not to ILC2 cells (25). This may partially explain the ILC plasticity observed in different tissues and under different conditions. Single-cell RNA sequencing of human ILC3 and ILC1 from the tonsils and intestine and bioinformatics analysis (RNA velocity) recently enabled the identification of a cluster of cells that represent a spectrum of cell conversions between ILC3 and ILC1, with a pronounced bias toward ILC1. Cell transfers into humanized mice demonstrated *in-vivo* conversion of ILC3 to ILC1, which occurred preferentially in TGF-β-expressing tissues, such as the spleen (26). As observed in mice, the formation of mature human ILC3 subsets not only occurs during fetal development but can also occur after birth locally in tissues, such as adult tonsils and intestine (19). Different developmental pathways and marked plasticity even in adult ILC complicate their classification into subtypes.

## 2 Literature survey results

**Figure 1:**
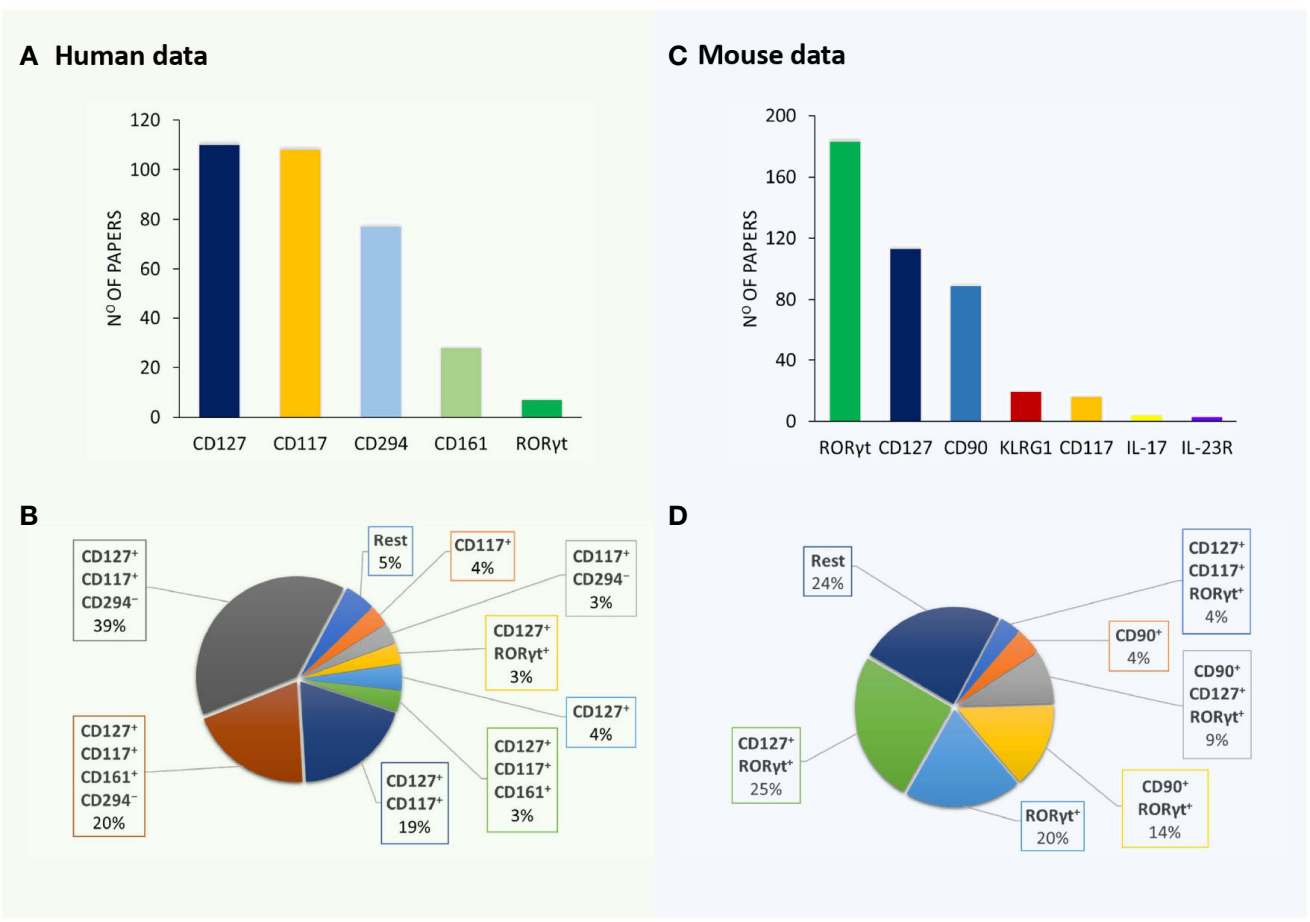
Markers and their combinations used for the identification of ILC3. Visual representation of data from **Tables 1**, **2**. The number of papers in which specific markers were used for the identification of human **(A)** and mouse **(B)** ILC3s. The most frequently used marker combinations for the identification of human **(C)** and mouse **(D)** ILC3s (% of all papers examined).

A detailed survey of papers on ILC3 was performed using the PubMed database (https://pubmed.ncbi.nlm.nih.gov/) until 15 June 2023. Searching for “ILC3 mouse” and “ILC3 human” retrieved more than 300 articles each. Papers containing data on ILC3 identification by flow cytometry (121 and 226 for human and mouse ILC3, respectively) were analyzed for the following information: phenotypic markers used for the definition of ILC3, lineage markers used for excluding immune cells other than ILC3, and organ/tissue of ILC3 origin. The obtained information was compiled in **Tables 1**, **2**, dedicated to human and mouse ILC3, respectively. The overview of the tables shows inconsistency in the markers used for the identification of ILC3, as well as of those used in lineage cocktails for the exclusion of non-ILC3 cells. The most commonly used inclusion markers for human ILC3 are CD117, CD127, and CD161, while CD294 is the most frequently used exclusion marker (**Figure 1A**). The most frequently used marker combinations for the identification of human ILC3 are 1) CD127^+^CD117^+^CD294^−^, 2) CD127^+^CD117^+^CD161^+^CD294^−^, and 3) CD127^+^CD117^+^ (**Figure 1B**). The most commonly used markers for mouse ILC3 are RORγt, CD127, and CD90 (**Figure 1C**), while the prevalent combinations are CD127^+^RORγt^+^ and CD90^+^RORγt^+^ (**Figure 1D**). A detailed list of the markers and their combinations is given in **Table 3**. Interestingly, RORγt is frequently used as a single marker for ILC3 identification in mice and is by far the predominantly used marker for ILC3 in mouse, but not in human samples (*vide infra*). The adequacy of the phenotypic markers used for defining ILC3 in relation to the tissue/organ of their origin is discussed in the following chapters.

**Table 1:** Phenotypic characterization of human ILC3.

Phenotype	Lineage	Tissue/organ	Reference
CD103^−^	CD3, CD19	Tonsils	(27)
CD117^+^	CD3, CD19, CD34, CD94	Tonsils	(28)
CD117^+^	CD94/NKG2A	UCB	(29)
CD117^+^CD294^−^	CD3, CD19, CD16, CD62L	Ileum	(30)
CD117^+^CD294^−^	CD3, CD1a, CD11c, CD14, CD19, CD34, CD123, TCRαβ, TCRγδ, CD303, FcεR1α, CD94	PB	(31)
CD117^+^RORγt^+^	CD94/NKG2A^−^	UCB	(32)
CD117^+^RORγt^+^	CD14, CD19, CD3, CD11b, CD11c, TCRγδ	Colon	(33)
CD127^+^	CD3	Intestine	(34)
CD127^+^	CD3	Colon LP	(35)
CD127^+^CD117^+^	CD1a, CD3, CD14, CD19, CD94, CD34, CD123, TCRαβ, TCRγδ, CD303, FcεR1α	Tonsils, intestine, mLN	(36)
CD127^+^CD117^+^	CD3, CD5, CD14, CD19, CD11c	Colon	(37)
CD127^+^CD117^+^	Data not found	Colon	(38)
CD127^+^CD117^+^	CD3, CD19, CD14, CD28, CD11c	PBMC	(39)
CD127^+^CD117^+^	CD19, CD11b, CD11c, CD3, CD5, CD14, FcεR1α,	Colon	(40)
CD127^+^CD117^+^	CD1a, CD3, CD14, CD19, CD20, CD203c	Nasal epithelium	(41)
CD127^+^CD117^+^	CD3, CD4, CD5, TCRαβ, TCRγδ, CD33, CD14, CD19, CD235a	mLN	(42)
CD127^+^CD117^+^CD294^−^	CD3, CD20, CD13, CD123, CD303, CD34, FcεR1α, CD11c	Colon LP, tonsils	(43)
CD127^+^CD117^+^CD161^+^	CD3, TCRαβ, TCRγδ, CD34, CD123, CD94, CD14, CD303, FcεR1α, CD1a, CD11c, CD19, B220	PB, UCB, tonsils	(44)
CD127^+^CD117^+^CD161^+^	CD3, CD4, CD19, CD20, CD14, CD34, CD11c, CD94	Tonsils, PB	(45)
CD127^+^CD117^+^CD161^+^CD294^−^	CD3, CD14, CD16, CD20, CD56	PB	(46)
CD127^+^CD117^+^CD161^+^CD294^−^	CD1a, CD3, CD14, CD19, CD34, CD94, CD123, FcεR1α, TCRαβ, TCRγδ, CD303	PBMC, tonsils	(47)
CD127^+^CD117^+^CD161^+^CD294^−^	CD1a, CD34, CD3, TCRαβ, TCRγδ, CD14, CD19, CD16, CD94, CD123, CD303, FcεR1α	PB	(48)
CD127^+^CD117^+^CD161^+^CD294^−^	CD3, CD4, CD14, CD16, CD19, CD21, CD94, CD11c, CD123, CD303	PBMC	(49)
CD127^+^CD117^+^CD161^+^CD294^−^	CD3, TCRαβ, TCRγδ, CD34, CD123, CD94, CD14, CD303, FcεR1α, CD1a, CD11c, CD19, B220	PBMC	(50)
CD127^+^CD117^+^CD161^+^CD294^−^	CD3, TCRαβ, TCRγδ, CD34, CD123, CD94, CD14, CD303, FcεR1α, CD1a, CD11c, CD19, B220	UCB	(51)
CD127^+^CD117^+^CD161^+^CD294^−^	CD3, CD19, CD20, CD14, CD11c	PB	(52)
CD127^+^CD117^+^CD161^+^CD294^−^	CD1a, CD3, CD4, CD8, CD14, CD16, CD19, CD34, CD94, CD303, TCRαβ, TCRγδ, FcεR1α	Thymus	(53)
CD127^+^CD117^+^CD161^+^CD294^−^	CD3, CD14, CD19, CD20, CD94, FcεR1α, TCRαβ, TCRγδ, CD123, CD34	Spleen, PBMC	(54)
CD127^+^CD117^+^CD161^+^CD294^−^	CD3	PBMC	(55)
CD127^+^CD117^+^CD294^−^	CD1a, CD3, CD11c, CD14, CD19, CD34, CD94, CD123, CD303, FcεR1α, TCRαβ, TCRγδ	PBMC, skin	(56)
CD127^+^CD117^+^CD294^−^	CD1a, CD3, CD4, CD5, CD14, CD19, CD16, CD34, CD94, CD123, CD303, TCRαβ, TCRγδ, FcεR1α	PB, UCB	(57)
CD127^+^CD117^+^CD294^−^	CD3, CD19, CD11b, CD11c	Bladder	(58)
CD127^+^CD117^+^CD294^−^	CD3, CD14, CD16, CD19, CD20, CD56	Abdominal fat, PBMC	(59)
CD127^+^CD117^+^CD294^−^	CD3, CD19	Distal ileum	(60)
CD127^+^CD117^+^CD294^−^	CD3, CD19, CD94, CD14, CD34, CD303	PB, tonsils	(61)
CD127^+^CD117^+^CD294^−^	CD3, CD4, CD14, CD16, CD19, CD8, CD15CD20, CD33, CD34, CD203c, FcεR1α	Lip tissue	(62)
CD127^+^CD117^+^CD294^−^	CD1a, CD8, CD19, CD3, CD4, CD14, CD16, CD34, CD123, TCRαβ, TCRγδ,	PB	(63)
CD127^+^CD117^+^CD294^−^	CD1a, CD3, CD14, CD11c, CD19, CD34, CD94, CD123, CD303, FcεR1α, TCRαβ, TCRγδ	Entheseal tissue	(64)
CD127^+^CD117^+^CD294^−^	CD34, CD3, CD19, FcεR1α, CD123, CD94, CD14, CD11c, CD1a, CD303	PBMC	(65)
CD127^+^CD117^+^CD294^−^	CD3, CD19, CD14, TCRαβ, TCRγδ, CD94, CD16, FcεR1α, CD34, CD123, CD303	PBMC	(66)
CD127^+^CD117^+^CD294^−^	CD34, CD3, CD19, FcεR1α, CD123, CD94, CD14, CD11c, CD1a, CD303	PB	(67)
CD127^+^CD117^+^CD294^−^	CD3, CD1a, CD14, CD19, TCRαβ, TCRγδ, CD1, CD303, FcεR1α, CD235α, CD66b, CD34, CD20, CD94	UCB, tonsils	(68)
CD127^+^CD117^+^CD294^−^	Data not found	PB	(69)
CD127^+^CD117^+^CD294^−^	CD3, CD14, CD19, CD11c, CD303a, CD15, CD203c, FcεR1α	PBMC	(70)
CD127^+^CD117^+^CD294^−^	CD1a, CD34, CD3, TCRαβ, TCRγδ, CD14, CD19, CD16, CD94, CD123, CD303, FcεR1α	PB	(71)
CD127^+^CD117^+^CD294^−^	CD3, CD4, CD8, CD14, CD15, CD16, CD19, CD20, CD33, CD34, CD203c, FcεR1α	PBMC	(72)
CD127^+^CD117^+^CD294^−^	CD3, CD19, CD94, CD1a, CD11c, CD123, CD303, CD14, FcεR1α, CD34	Tonsils	(73)
CD127^+^CD117^+^CD294^−^	CD1a, CD3, CD4, CD56, CD16, CD11c, CD14, CD19, CD94, CD34, CD123, TCRαβ, TCRγδ, CD303, FcεR1α	PB	(74)
CD127^+^CD117^+^CD294^−^	CD3	PB	(75)
CD127^+^CD294^−^	CD3, CD19, CD14, CD94	Tonsils	(19)
CD127^+^IL-23R^+^	CD2, CD3, CD14, CD16, CD19, CD56, CD235a	PBMC	(76)
CD127^+^RORγt^+^	CD15, CD14, CD3, CD19, CD56, CD11b	Decidua	(77)
CD300LF^+^CD196^+^	CD3, CD19	Tonsils	(26)
CD336^+^	CD3, CD14, CD19, CD20, CD94	UCB	(78)
CD336^+^CD56^+^	CD3, CD1a, CD14, CD19, TCRαβ, TCRγδ, CD123, CD303, FcεR1α, CD235a, CD66b, NKG2A	UCB	(79)
CD45^+^	CD3, CD4, CD8, CD56, CD14, CD19, TCRγδ	Ileum	(80)
CD45^+^CD117^+^	CD3, CD5, CD14, FcεR1α, CD11b, CD11c, CD19	Colon LP	(13)
CD45^+^CD117^+^	CD3, CD14, CD19, CD56, CD34, CD11a, CD94	UCB	(81)
CD45^+^CD117^+^CD294^−^	TCRαβ, TCRγδ, CD19, CD94, CD1a, CD123, CD14, CD303, FcεR1α, CD34	PBMC, ileum/colon	(82)
CD45^+^CD117^+^CD294^–^	CD3, CD19, CD20	PB, skin	(83)
CD45^+^CD127^+^	CD19, CD14, CD3	Decidua, tonsils, PBMC	(84)
CD45^+^CD127^+^	CD3, CD14, CD19, CD20, CD123, CD141, FcεR1α	Tonsils, colon	(85)
CD45^+^CD127^+^	CD3, CD14, CD16, CD19, CD20, CD56	PBMC	(86)
CD45^+^CD127^+^ CD117^+^CD294^−^	CD3, CD5, CD11c, CD16, CD19, TCRαβ	PBMC	(87)
CD45^+^CD127^+^CD117^+^CD294^−^	CD3, CD5, CD11b, CD11c, CD14, CD16, CD19, CD34, CD123, FcεR1α	PBMC	(88)
CD45^+^CD127^+^ CD117^+^CD294^−^	CD1a, CD3, CD4, CD14, CD16, CD19, CD34, CD303, FcεR1α	PB	(89)
CD45^+^CD127^+^CD117^+^	CD3, CD14, CD19, CD34	Inguinal LN	(90)
CD45^+^CD127^+^CD117^+^	CD3ε, CD11c, CD11b, CD14, CD19, CD49b, FcεR1α	Sputum, PB	(91)
CD45^+^CD127^+^CD117^+^	CD34, CD19, CD20, CD3, CD16, CD14, CD11c, CD123	PB	(92)
CD45^+^CD127^+^CD117^+^	CD3, CD19, CD11c, CD11b	Lung	(93)
CD45^+^CD127^+^CD117^+^	CD3, CD14, CD19, CD20	Duodenum	(94)
CD45^+^CD127^+^CD117^+^	CD19, CD3, CD1a, CD11b, CD34, FcεR1α, CD14, CD11c, CD94	Distal ileum	(95)
CD45^+^CD127^+^CD117^+^	CD3, CD11b, CD11c, CD14, CD19, CD34, CD94, CD123, FcεR1α	Tonsils, intestine	(96)
CD45^+^CD127^+^CD117^+^	CD3, CD19, CD94, CD14, CD16, CD1a, CD303, CD123, FcεR1α, CD34	Periodontal ligament tissues	(97)
CD45^+^CD127^+^CD117^+^	CD3, CD11c, CD14, CD19, CD34, CD94, CD123, FcεR1α	Colon	(98)
CD45^+^CD127^+^CD117^+^	CD1a, CD3, CD11c, CD14, CD19, CD94, CD34, CD123, TCRαβ, TCRγδ, CD303, FcεR1α	Tonsils, LP	(99)
CD45^+^CD127^+^CD117^+^	CD11b, CD11c, B220, CD3ε, CD5, CD8α	Terminal ileum	(100)
CD45^+^CD127^+^CD117^+^	CD34, CD19, CD14, CD3, CD94	Decidua	(101)
CD45^+^CD127^+^CD117^+^	CD34, CD14, CD19, CD3, CD94	Decidua	(102)
CD45^+^CD127^+^CD117^+^	CD19, CD14, CD3, CD94	Decidua	(103)
CD45^+^CD127^+^CD117^+^	CD3, CD4, CD5, CD14 CD16, CD19, TCRαβ, TCRγδ, CD94, NKG2A	PB	(104)
CD45^+^CD127^+^CD117^+^CD161^+^	CD3, TCRαβ, TCRγδ, CD14, CD16, CD19, CD94, FcεR1α, CD123, CD303	Tonsils	(105)
CD45^+^CD127^+^CD117^+^CD161^+^	CD3, CD19	Colonic mucosa, PBMC	(106)
CD45^+^CD127^+^CD117^+^CD161^+^CD294^−^	CD14, CD1a, CD3, CD34, CD123, FcεR1α, TCRαβ, TCRγδ, CD303, CD19	Tonsils, ileum/colon	(107)
CD45^+^CD127^+^CD117^+^CD161^+^CD294^−^	CD3	PB	(108)
CD45^+^CD127^+^CD117^+^CD161^+^CD294^−^	CD3, CD14, FcR1α, CD34, CD123, CD1a, TCRαβ, TCRγδ, CD94, CD303, CD19	Tonsils	(109)
CD45^+^CD127^+^CD117^+^CD161^+^CD294^−^	CD1a, CD3, CD14, CD16, CD19, CD34, CD94, CD123, CD303, FcεR1α, TCRαβ, TCRγδ,	Skin, PBMC	(110)
CD45^+^CD127^+^CD117^+^CD161^+^CD294^−^	CD1a, CD3, CD4, CD8, CD14, CD16, CD19, CD34, CD94, CD123, CD303, FcεR1α, TCRαβ, TCRγδ	PBMC, tonsils, UCB, bone marrow	(111)
CD45^+^CD127^+^CD117^+^CD161^+^CD294^−^	CD1a, CD3, CD14, CD19, FcεR1α, CD34, CD123, CD94, CD303, TCRαβ, TCRγδ	Tonsils, PBMC	(112)
CD45^+^CD127^+^CD117^+^CD161^+^CD294^−^	CD1a, CD3, CD11c, CD14, CD16, CD19, CD34, CD123, FcεR1α, NKp80, CD303, TCRαβ, TCRγδ	Intestine, colon, PB	(113)
CD45^+^CD127^+^CD117^+^CD161^+^CD294^−^	CD1a, CD4, TCRαβ, TCRγδ, CD3, CD11c, CD14, CD94, CD19, CD123, CD303, CD34, FcεR1α, CD16	PB	(114)
CD45^+^CD127^+^CD117^+^CD161^+^CD294^−^	CD3, CD4, CD19, CD94, CD1a, CD11c, CD123, BDAC2, CD14, CD34, FcεR1α	PBMC	(115)
CD45^+^CD127^+^CD117^+^CD161^+^CD294^−^	CD11c, CD123, CD14, CD19, CD1a, CD3, CD303a, CD94, FcεR1α, TCRαβ, TCRγδ, CD34	Colon, PBMC	(116)
CD45^+^CD127^+^CD117^+^CD161^+^CD294^−^	Without	PB	(117)
CD45^+^CD127^+^CD117^+^CD161^+^CD294^−^	CD3, CD4, CD19, CD11b	PBMC	(118)
CD45^+^CD127^+^CD117^+^CD161^+^CD294^−^	CD3, CD45CD19, CD14, CD1a, CD94, CD34	PB	(119)
CD45^+^CD127^+^CD117^+^CD161^+^CD294^−^	CD3, CD1CD11c, CD34, CD123, TCRγδ, TCRαδ, CD303, FcεR1α, CD19, CD14, CD94	PB, UCB, placenta	(120)
CD45^+^CD127^+^CD117^+^CD294^−^	CD3, CD4, CD8, CD11c, CD14, CD19, CD34, CD94, CD123, FcεR1α	Ileum, colon	(121)
CD45^+^CD127^+^CD117^+^CD294^−^	CD3, CD14, CD19, CD11b, CD11c, CD49b, FcεR1α	Terminal ileum, colon	(122)
CD45^+^CD127^+^CD117^+^CD294^−^	CD3, CD16, CD19, CD20, CD14, CD56	Thymus, PB	(123)
CD45^+^CD127^+^CD117^+^CD294^−^	CD3, CD11c, CD14, CD16, CD19, CD20, CD34, CD56, CD94, FcεR1α	PB	(124)
CD45^+^CD127^+^CD117^+^CD294^−^	CD3, CD19, CD11c, CD11b, CD14	Ileal LP, PBMC	(125)
CD45^+^CD127^+^CD117^+^CD294^−^	CD3, CD11c, CD14, CD16, CD19, CD34	PBMC	(126)
CD45^+^CD127^+^CD117^+^CD294^−^	CD3, CD5, CD11b, CD11c, CD19, FcεR1α	Intestine	(127)
CD45^+^CD127^+^CD117^+^CD294^−^	CD3, CD14, CD16, CD19, CD20, CD56	PB	(128)
CD45^+^CD127^+^CD117^+^CD294^−^	CD3, CD14, CD19, CD303, CD123, CD1a, TCRαβ, TCRγδ, CD34, FcεR1α,	PB	(129)
CD45^+^CD127^+^CD117^+^CD294^−^	CD3, CD19, CD14, CD34, CD94, CD123, TCRαβ, TCRγδ, FcεR1α	Liver, PB	(130)
CD45^+^CD127^+^CD117^+^CD294^−^	CD3, CD14, CD16, CD19, CD20, CD56	PB	(131)
CD45^+^CD127^+^CD117^+^CD294^−^	TCRγδ, TCRαβ, CD3, CD19, CD14, CD16, CD94, CD123, CD34, CD303, FcεR1α	Intestine, PB	(132)
CD45^+^CD127^+^CD117^+^CD294^−^	Data not found	PBMC	(133)
CD45^+^CD127^+^CD117^+^CD294^−^	CD3, CD14, CD19, CD20	Terminal ileum LP, PBMC	(134)
CD45^+^CD127^+^CD117^+^CD294^−^	CD3, CD14, CD19, CD94	PB	(135)
CD45^+^CD127^+^CD117^+^CD294^−^	CD3, CD14, CD16, CD19, CD20, CD56	PBMC	(136)
CD45^+^CD127^+^CD117^+^CD294^−^	CD3, CD11c, CD14, CD16, CD19, CD20, CD34, CD56, CD94, FcεR1α	PBMC	(137)
CD45^+^CD127^+^CD117^+^CD294^−^	CD14, CD16, CD19, FcεR1α, CD3, CD34, CD123, CD1a, CD303, TCRαβ, TCRγδ, CD94	Tonsils	(138)
CD45^+^CD127^+^CD117^+^CD294^−^	CD11b, CD14, CD16, CD303a, TCRαβ, TCRγδ, CD19, CD123, CD34, FcεR1α, CD1a, CD94, CD3, CD20, CD11c	PB	(139)
CD45^+^CD127^+^CD117^+^CD294^−^	CD3, CD19, CD14, CD34, CD94	LN, spleen, tonsils, PB	(140)
CD45^+^CD127^+^CD117^+^CD294^−^	CD1a, CD3, CD4, CD11c, CD14, CD19, CD34, CD123, TCRαβ, TCRγδ, CD303, FcεR1α, CD94	PB, skin	(141)
CD45^+^CD127^+^CD117^+^ CD294^−^	CD3, CD19, CD14, CD11c, CD123, CD34, FcεR1α, CD94, TCRαβ	PB	(142)
CD45^+^CD127^+^CD117^+^CD294^−^	CD3, CD19, CD14, CD11c, CD123, CD34, FcεR1α, TCRαβ, CD94	PB	(143)
CD45^+^CD127^+^CD117^+^CD294^–^	CD3, CD11c, CD14, CD19, CD34, CD94, CD123, FcεR1α	PB, peritoneal fluid, endometrium	(144)
CD45^+^CD127^+^CD294^−^	CD3, TCRαβ, TCRγδ, CD14, CD19, CD20, CD94, NKp80, CD16, CD1a, CD12, FcεR1α, BDCA^−^2	Liver, tonsils, colon, blood	(145)
CD45^+^CD127^+^RORγt^+^	CD3, TCRαβ, CD20, CD11c, CD11b, CD123, CD303, CD14, FcεR1α, CD31, CD34.	Endomyocardium	(146)
CD45^+^CD127^+^RORγt^+^	CD3, CD5, CD19	Cerebrospinal fluid	(147)
CD45^+^CD127^+^RORγt^+^	CD3, CD5, CD11b, CD11c, CD14, FcεR1α	BALF, lungs	(148)
RORγt^+^	CD3, CD14, CD16, CD19, CD20, CD56	PB	(149)

BALF, bronchoalveolar fluid; LN, lymph node; LP, lamina propria; mLN, mesenteric lymph node; PB, peripheral blood; PBMC, peripheral blood mononuclear cell; UCB, umbilical cord blood.

**Table 2:** Phenotypic characterization of mouse ILC3.

Phenotype	Lineage	Tissue/organ	Reference
CD117^+^CD4^+^	CD3, CD19	PBMC	(150)
CD117^+^RORγt^+^	Data not found	SI LP	(151)
CD117^+^RORγt^+^	CD3, CD8α, CD11c, CD19, B220, Ly6G/6C, TCRβ, TCRγδ, NK1.1	Spleen	(152)
CD127^+^CD117^+^	CD3, B220, CD11b, Ter119, Ly6G	Aorta, spleen	(153)
CD127^+^CD117^+^RORγt^+^	Data not found	Fetal spleen	(154)
CD127^+^CD117^+^RORγt^+^	CD3	Neonatal SI LP	(155)
CD127^+^CD117^+^RORγt^+^	NK1.1	PL	(156)
CD127^+^Id2^+^	CD3, CD19, CD4, CD5, CD8, TCRβ, TCRγδ, NK1.1, CD11b, Ly6G/6C, CD11c, Ter119, NK1.1, Bcl11b	Lung	(157)
CD127^+^IL-22^+^	CD3	SI LP	(158)
CD127^+^KLRG^−^	CD19, CD3, CD11c, CD25, Ly6G/6C	Lung	(159)
CD127^+^RORγt^+^	CD3	SI LP	(160)
CD127^+^RORγt^+^	CD3ε, CD5, CD19, B220, Ly6G/6C	SI LP	(161)
CD127^+^RORγt^+^	CD3, B220, CD5, NK1.1, CD11b, CD11c	Colon, mLN	(40)
CD127^+^RORγt^+^	CD3, B220, CD11b, Ly6G, Ter119	Skin	(162)
CD127^+^RORγt^+^	CD3, B220, CD11c, CD11b, CD5	Ear skin, auricular LN, SI LP, lung, mLN	(163)
CD127^+^RORγt^+^	CD3ε, CD5, CD19, B220, CD11b, CD11c, Ter119, F4/80, Ly6G/6C, CD49b, FcεR1α	Liver, spleen	(65)
CD127^+^RORγt^+^	Data not found	SI LP	(164)
CD127^+^RORγt^+^	CD3ε, CD8α, CD19, B220, CD11c, CD11b, Ter119, Ly6G/6C, TCRβ, TCRγδ, NK1.1	Blood	(165)
CD127^+^RORγt^+^	TCRγδ, CD3ε, CD19, CD5, Ly6G/6C, Ter119	SI LP	(166)
CD127^+^RORγt^+^	CD3, CD19	SI	(167)
CD127^+^RORγt^+^	CD3, B220, CD11b, Ter119, Ly6G/6C, CD11c, NK1.1, CD8α, CD4	Spleen	(168)
CD127^+^RORγt^+^	Data not found	Blood, spleen, kidney	(169)
CD127^+^RORγt^+^	B220, CD3, CD5, CD11b, CD11c, CD19, CD49b, CD123, F4/80, FcεR1α, Ly6G/6C, Ter119, CD8α, CD3i	Thymus, mLN	(170)
CD127^+^RORγt^+^	Data not found	Lung	(171)
CD127^+^RORγt^+^KLRG1^−^	CD3, CD5, CD11b, CD11c, B220	SI	(172)
CD127^+^RORγt^+^	CD3, CD8α, CD19, Ly6G/6C	SI LP	(173)
CD45^+^CD117^+^RORγt^+^	CD3, Ly6G/6C, CD11b, B220, Ter119	Colon	(174)
CD45^+^CD117^+^RORγt^+^	CD3, CD8, CD11b, CD19, MHC II, F4/80, CD161, Ly6G, F4/80	Neonatal lung	(175)
CD45^+^CD127^+^	CD3, NK1.1	SI LP	(176)
CD45^+^CD127^+^	CD3, CD19, CD8α, CD11b, CD4, NK1.1	SI	(177)
CD45^+^CD127^+^	Data not found	Colon LP	(178)
CD45^+^CD127^+^	NK1.1, other data not found	SI LP	(179)
CD45^+^CD127^+^CD117^+^	CD3, CD19, CD11c, CD11b, Ly6G/6C, NK1.1	SI LP	(180)
CD45^+^CD127^+^CD117^+^	Data not found	Colon, SI	(181)
CD45^+^CD127^+^CD117^+^	CD3, CD19, B220, CD11b, Ly6G/6C, CD11c, TCRβ, TCRγδ	Colon LP	(182)
CD45^+^CD127^+^CD117^+^RORγt^+^	CD3ε, CD11b, B220, Ter119, Ly6G/6C, ICOS	Lung	(183)
CD45^+^CD127^+^CD117^+^RORγt^+^	CD3ε, CD11b, B220, Ter119, Ly6G/6C	SI LP	(184)
CD45^+^CD127^+^CD117^+^CD4^+^RORγt^+^	CD3, other data not found	LP	(185)
CD45^+^CD127^+^CD117^+^RORγt^+^IL-23R^+^	CD3, CD19, CD11c, Ly6G, F4/80, CD14, NK1.1	PL, LP, spleen	(186)
CD45^+^CD127^+^CD25^+^	CD3, CD5, CD8, NK1.1, B220, CD11c, CD11b	mLN	(13)
CD45^+^CD127^+^CD4^+^	CD3	Spleen	(187)
CD45^+^CD127^+^RORγ^+^	NK1.1, other data not found	Pooled spleen, lung, intestine, peripheral LN	(188)
CD45^+^CD127^+^RORγt	CD3, Ly6G/6C, CD11b, B220, Ter119	Lungs	(189)
CD45^+^CD127^+^RORγt^+^	CD8, CD3ε, TCRβ, TCRγδ, B220, Ter119, Ly6G/6C, NK1.1	Bladder, kidney, SI	(190)
CD45^+^CD127^+^RORγt^+^	CD3, B220, CD11b, Ter119, Ly6G/6C	SI	(191)
CD45^+^CD127^+^RORγt^+^	ICOS, other data not found	Lung	(192)
CD45^+^CD127^+^RORγt^+^	Data not found	Colon LP	(193)
CD45^+^CD127^+^RORγt^+^	CD3ε, TCRβ, CD19	Colon	(194)
CD45^+^CD127^+^RORγt^+^	CD19, CD3, CD5, F4/80, FcεR1α, Ly6G/6C	SI	(195)
CD45^+^CD127^+^RORγt^+^	CD3ε, B220, Ly6G/6C, CD11c, CD11b, Ter119, FcεR1α	Liver	(196)
CD45^+^CD127^+^RORγt^+^	CD3, CD11b, CD11c, B220, Ter119, FcεR1α, T-bet	SI	(197)
CD45^+^CD127^+^RORγt^+^	Data not found	Colon LP	(198)
CD45^+^CD127^+^RORγt^+^	CD3, NK1.1, CD11b, Ter119, Ly6G/6C, CD11c, B220	Colon LP	(199)
CD45^+^CD127^+^RORγt^+^	CD3ε, CD5, CD8α, NK1.1, CD11c, CD11b, B220	SI LP	(127)
CD45^+^CD127^+^RORγt^+^	CD3, CD19, Ly6G/6C	Meninges	(200)
CD45^+^CD127^+^RORγt^+^	CD3, CD19	Colon LP, SI LP PP	(201)
CD45^+^CD127^+^RORγt^+^	CD3	SI LP	(202)
CD45^+^CD127^+^RORγt^+^	Data not found	SI LP	(203)
CD45^+^CD127^+^RORγt^+^	CD3, CD4, CD19, NK1.1, CD11b, CD11c, Ly6G/6C, F4/80, Ter119	SI LP	(204)
CD45^+^CD127^+^RORγt^+^	TCRβ, TCRδ, CD19, CD11c, CD11b, Ly6G/6C, Ter119	SI LP	(205)
CD45^+^CD127^+^RORγt^+^	B220, CD3, CD5, CD11b, CD11c	mLN	(206)
CD45^+^CD127^+^RORγt^+^	CD3, CD19	SI LP	(207)
CD45^+^CD127^+^RORγt^+^	CD3	SI LP	(100)
CD45^+^CD127^+^RORγt^+^	CD3ε, CD5, F4/80, CD11b, CD19, Ly6G	Lung, bone marrow, SI LP	(148)
CD45^+^CD127^+^RORγt^+^	CD3	SI LP, colon LP	(208)
CD45^+^CD127^+^RORγt^+^	CD3, CD4, CD5, CD11b, CD11c, CD19, B220, F4/80, FcεR1α, Ly6G/6C, TCRβ, TCRγδ, Ter119	Lung, intestine	(209)
CD45^+^CD127^+^RORγt^+^	CD3, B220, CD11b, Ter119, Ly6G/6C	SI LP	(210)
CD45^+^CD127^+^RORγt^+^	CD3, CD19, CD11c	Spleen	(210)
CD45^+^CD127^+^RORγt^+^	CD3, B220, CD19, CD11b, Ter119, Ly6G/6C, CD5, FcεR1α	Colon LP, SI LP	(211)
CD45^+^CD127^+^RORγt^+^	CD3, B220, CD19, CD11b, Ter119, Ly6G/6C, CD5, FcεR1α	Colon LP, SI LP	(212)
CD45^+^CD127^+^RORγt^+^	CD3, Ly6G/6C, CD11b, B220, Ter119	Lung	(213)
CD45^+^CD127^+^RORγt^+^	CD3, CD4, CD14, CD16, CD19, CD8, CD15, CD20, CD34, CD203	Meninges	(147)
CD45^+^CD127^+^RORγt^+^	CD3, CD19	SI	(214)
CD45^+^CD127^+^RORγt^+^	CD3, CD19, NK1.1, CD11b	Liver	(215)
CD45^+^CD127^+^RORγt^+^	CD3	SI	(216)
CD45^+^CD127^+^RORγt^+^	CD11b, CD3ε, B220, SiglecF, FcεR1α	Lung	(217)
CD45^+^CD127^+^RORγt^+^	CD3, CD4, CD11b, CD11c, CD19, CD49b, F4/80, FcεR1α	Colon	(122)
CD45^+^CD127^+^RORγt^+^	Data not found	mLN, inguinal LN	(218)
CD45^+^CD127^+^RORγt^+^KLRG1^−^	CD5, CD8α, CD3, Ly6G/6C, TCRγδ, FcεR1α, CD19, CD11c, NK1.1	SI LP	(219)
CD45^+^CD127^+^RORγt^+^	CD3, CD4, CD19, CD11b, CD11c, Ly6G/6C, F4/80, Ter119	SI LP	(220)
CD45^+^CD127^+^RORγt^+^	CD3, CD19	Colon LP, SI LP	(221)
CD45^+^CD127^+^RORγt^+^	CD3, CD11b, B220, Ly6G/6C, Ter119	SI LP	(222)
CD45^+^CD127^+^RORγt^+^	CD3ε, CD11b, B220, Ly6G/6C, Ter119	Colon LP	(223)
CD45^+^CD127^+^RORγt^+^	CD3ε, CD11b, B220, Ter119, Ly6G/6C, CD49b	LN, spleen, lung	(224)
CD45^+^CD90^+^	CD11b, CD11c, B220, Ly6G/6C, FcεR1α, Ter119	Skin	(225)
CD45^+^CD90^+^	CD3, CD5, CD19, B220	SI LP, colon LP	(226)
CD45^+^CD90^+^	CD3ε, CD5, CD19, CD11b, CD11c, NK1.1, KLRG1	SI	(227)
CD45^+^CD90^+^	NK1.1	SI LP	(202)
CD45^+^CD90^+^	CD3ε, CD11b, CD45R, TER119, Ly6GLy6C	PP	(228)
CD45^+^CD90^+^	CD3, CD5, CD19, B220, Ly6G, FcεR1α, CD11c, CD11b, Ter119, NK1.1, CD16/CD32	SI LP, colon LP	(229)
CD45^+^CD90^+^	CD3	Colon LP	(230)
CD45^+^CD90^+^	CD3ε, CD5, CD19	SI LP	(231)
CD45^+^CD90^+^KLRG1^−^	CD8, CD11b, CD11c, CD19, NK1.1, Ly6G/6C, Ter119, CD3ε	mLN	(232)
CD45^+^CD90^+^CD127^+^	Data not found	mLN, inguinal LN	(218)
CD45^+^CD90^+^CD127^+^	CD11b, CD3ε, CD5, CD11c, CD19, NK1.1, Ly6G/6C, Ter119, TCRγδ	Lung	(233)
CD45^+^CD90^+^CD127^+^	CD3, CD4, CD5, CD8, CD11b, CD11c, CD19, CD49b, Ly6G/6C, NK1.1	Spleen, mLN, PP, SI, colon	(234)
CD45^+^CD90^+^CD127^+^	CD3, CD19	SI LP	(235)
CD45^+^CD90^+^CD127^+^KLRG1^−^	CD3ε, CD5, CD8α, NK1.1, CD11c, CD11b, B220, CD27	SI LP	(127)
CD45^+^CD90^+^CD127^+^RORγt^+^	CD3ε, CD4, CD8, TCRγδ, CD11b, CD11c, CD19, B220, Ly6G/6C, NK1.1, Ter119.	Spleen, mLN, SI LP, colon, lung, liver, bone marrow	(236)
CD45^+^CD90^+^CD127^+^RORγt^+^	CD19, CD3, CD11b, Ly6G/6C, CD11c	Spleen	(237)
CD45^+^CD90^+^CD127^+^RORγt^+^	CD11b, CD11c, B220, CD3ε, CD5, CD8α	SI LP, colon LP	(100)
CD45^+^CD90^+^CD127^+^RORγt^+^	CD11b, CD11c, CD19, CD3ε, CD5, CD8α	mLN, spleen, PP	(238)
CD45^+^CD90^+^CD127^+^RORγt^+^KLRG1^−^	CD3, CD5, CD19, B220, Ly6G/6C, NK1.1, CD11b, CD11c	Intestine, mLN	(98)
CD45^+^CD90^+^CD127^+^RORγt^+^KLRG1^−^	CD3ε, CD49b, CD11b, CD94, CD5, TCRγδ, CD19, Ter119, Ly6G/6C, CD45RB, Ly6G	Lung	(239)
CD45^+^CD90^+^CD127^+^RORγt^+^KLRG1^−^	CD3ε, CD5, CD8α, NK1.1, TCRγδ, CD11b, CD11c, B220	SI, colon	(240)
CD45^+^CD90^+^CD127^+^CD117^+^RORγt^+^KLRG1^−^	CD3ε, TCRβ, CD19, B220, Ly6G/6C, CD11b	SI LP, lung, mLN	(241)
CD45^+^CD90^+^CD127^+^KLRG1^−^	B220, CD11c, NK1.1	Spleen, colon LP	(242)
CD45^+^CD90^+^CD127^+^RORγt^+^	CD3, CD5, NKK1.1, B220, CD11b, CD11c	mLN, SI LP	(243)
CD45^+^CD90^+^CD127^+^RORγt^+^	Ter119, F4/80, CD11b, CD11c, FcεR1α, Ly6G/6C, CD19, CD3, TCRβ, TCRγδ	SI LP, skin	(244)
CD45^+^CD90^+^CD127^+^RORγt^+^	CD3, B220, CD11b, CD11c Ter119, Ly6G/6C, TCRγδ	Bladder	(58)
CD45^+^CD90^+^CD127^+^RORγt^+^	CD3ε, CD5, FcεR1α, F4/80, CD11b, CD11c, B220	mLN, SI LP, PP	(125)
CD45^+^CD90^+^CD127^+^RORγt^+^	CD3e, CD11b, Ter119, B220, Ly6G/6C	Colon LP	(245)
CD45^+^CD90^+^CD127^+^RORγt^+^	CD3ε, CD8α, TCRβ, TCRγδ, CD19, Ly6G/6C, CD11c, Ter119	Colon LP	(246)
CD45^+^CD90^+^CD127^+^RORγt^+^KLRG1^−^	CD3, CD19, NK1.1	SI LP	(247)
CD45^+^CD90^+^IL-17^+^	B220, CD11b, CD11c, Ly6G/6C, NK1.1	Skin	(248)
CD45^+^CD90^+^IL-23R^+^	CD3, NK1.1, CD11b	Ileum LP	(50)
CD45^+^CD90^+^KLRG1^−^	CD3ε, CD8α, CD19, B220, CD11c, CD11b, Ter119, Ly6G/6C, TCRβ, TCRγδ, NK1.1	SI LP, mLN, spleen, lung	(165)
CD45^+^CD90^+^KLRG1^−^	CD3, Ly6G/6C, CD11b, B220, Ter119, NK1.1	Ileum LP, colon LP, mLN, spleen	(249)
CD45^+^CD90^+^RORγt^+^	CD4, CD8, CD11b, CD11c, CD19, B220, NK1.1, Ter119, Ly6G/6C, FcεR1α, TCRβ, TCRγ, CD3ε	Skin	(250)
CD45^+^CD90^+^RORγt^+^	CD3, Ly6G/6C, CD11b, B220, Ter119	SI LP	(251)
CD45^+^CD90^+^RORγt^+^	CD3, B220, CD11c, CD11b, NK1.1	Colon LP, spleen, SI LP	(252)
CD45^+^CD90^+^RORγt^+^	CD3, other data not found	SI LP, ileal LP	(253)
CD45^+^CD90^+^RORγt^+^	B220, CD3, NK1.1, CD11b	SI LP, blood, inguinal LN, spleen	(44)
CD45^+^CD90^+^RORγt^+^	CD3	Colon, SI, skin, lung	(254)
CD45^+^CD90^+^RORγt^+^	CD3, CD19	SI LP, colon LP	(255)
CD45^+^CD90^+^RORγt^+^	Data not found	SI LP, colon LP, cecum	(256)
CD45^+^CD90^+^RORγt^+^	CD11b, CD11c, CD19	SI LP	(257)
CD45^+^CD90^+^RORγt^+^	CD3, CD5, CD8α, CD11b, B220, NK1.1	mLN, PP, SI LP	(258)
CD45^+^CD90^+^RORγt^+^	CD3, CD49b, TCRβ, TCRγδ, CD5, F4/80, CD11c, Ly6G/6C, CD19, FcεR1α, B220, CD27	Lung	(259)
CD45^+^CD90^+^RORγt^+^	CD3ε, CD11b, CD11c, F4/80, Ly6G/6C, Ter119, B220	SI LP	(260)
CD45^+^CD90^+^RORγt^+^	NK1.1	Bone marrow	(235)
CD45^+^CD90^+^RORγt^+^	CD3, TCRβ, CD5, CD19, CD11b, CD11c, Ly6G/6C, FcεR1α, CD31, Ter119	Heart	(146)
CD45^+^CD90^+^RORγt^+^	CD11b, CD11c, B220, CD4, TCRβ	Lung	(261)
CD45^+^CD90^+^RORγt^+^	Without	SI LP, colon LP, mLN	(262)
CD45^+^CD90^+^RORγt^+^	Data not found	Colon LP	(262)
CD45^+^CD90^+^RORγt^+^	Ter119, CD11b, Ly6G/6C, CD3ε, B220, TCRβ, NK1.1	Colon LP	(263)
CD45^+^CD90^+^RORγt^+^	CD4, TCRβ, TCRγδ, CD8α, CD8β, CD19, CD11b, CD11c, DX5, Ly6G/6C, Ter119, NK1.1	SI LP, colon LP	(264)
CD45^+^CD90^+^RORγt^+^	Without	SI LP	(265)
CD45^+^CD90^+^RORγt^+^	CD11c, CD11b, NK1.1, B220, CD3	Colon LP	(266)
CD45^+^CD90^+^RORγt^+^	CD3, CD19, B220, CD11c, Ly6G/6C	SI LP	(267)
CD45^+^CD90^+^RORγt^+^	CD3	Colon LP	(268)
CD45^+^CD90^+^RORγt^+^	CD3, CD5, CD19, CD11c, CD11b	SI	(269)
CD45^+^CD90^+^RORγt^+^	CD3ε, Ly6G/6C, CD11b, B220, Ter119	SI LP, colon LP, lung, liver, PP, skin	(270)
CD45^+^CD90^+^RORγt^+^	Data not found	Colon, SI	(271)
CD45^+^CD90^+^RORγt^+^	CD3ε, CD5, CD19	SI	(272)
CD45^+^CD90^+^RORγt^+^	CD3	SI LP	(273)
CD45^+^CD90^+^RORγt^+^ KLRG1^−^	Ly6G/6C, CD3ε, CD11b, B220	Liver	(274)
CD45^+^CD90^+^RORγt^+^	Data not found	SI	(275)
CD45^+^CD90^+^RORγt^+^KLRG1^−^	CD3, B220, CD49a, CD11b, CD11c, NK1.1	SI	(128)
CD45^+^CD90^+^RORγt^+^KLRG1^−^	CD11c	Colon LP, SI	(242)
CD45^+^CD90^+^RORγt^+^KLRG1^−^	CD3, Ly6G/6C, CD11b, B220, Ter119, NK1.1	Colon LP, SI LP, mLN, spleen, PP	(276)
CD45^+^CD90^+^Sca-1^hi^	CD3, B220, CD11b, Ly6G/6C, Ter119	Neonatal SI, colon, mLN, spleen	(277)
CD45^+^CD90^hi^RORγt^+^	CD3, Ly6G/6C, CD11b, B220, Ter119	SI, colon	(278)
CD45^+^IL-17^+^	CD3, CD19, CD11b, CD11c, CD49b, F4/80, FcεR1α	Lung	(279)
CD45^+^IL-17^+^	TCRγδ, TCRβ	Spleen	(280)
CD45^+^IL-17^+^IL-22^+^	CD5, CD11b, B220, Ly6G/6C, Ter119, CD3	Colon LP	(281)
CD45^+^RORγt^+^	CD3ε, CD19	Liver	(282)
CD45^+^RORγt^+^	TCRαβ, TCRγδ, CD5, CD3ε, CD19, Ly6G/6C, F4/80	Colon LP, SI LP	(283)
CD45^+^RORγt^+^	CD3, CD11b, B220, Ly6G, Ter119	Colon LP	(284)
CD45^+^RORγt^+^	Data not found	Colon LP	(285)
CD45^+^RORγt^+^	CD3, CD19	SI LP	(286)
CD45^+^RORγt^+^	CD3, CD11b, B220, Ly6G, Ter119	Colon, mLN	(287)
CD45^+^RORγt^+^	CD3, CD19	SI LP	(288)
CD45^+^RORγt^+^	CD3, CD19	SI, colon, cecum	(289)
CD45^+^RORγt^+^	CD3, CD19	SI LP	(290)
CD45^+^RORγt^+^	CD3	SI LP	(291)
CD45^+^RORγt^+^	CD3, CD19	SI LP, colon LP	(292)
CD45^+^RORγt^+^	CD3, B220, Ter119, Ly6G/6C, CD11b, CD19, NK1.1	SI LP	(293)
CD45^+^RORγt^+^	CD3, CD19	SI LP	(294)
CD45^+^RORγt^+^	TCRβ, B220, TCRγd	mLN, jejunum LP, colon LP, cecum	(295)
CD45^+^RORγt^+^	Data not found	SI LP, colon LP	(296)
CD45^+^RORγt^+^	NK1.1, other data not found	Colon LP	(297)
CD45^+^RORγt^+^	CD3, CD19, B220, Ly6G/6C, CD11c, CD11b	Pooled axillary, brachial, inguinal and cervical LN, mLN	(298)
CD45^+^RORγt^+^	Data not found	Ileum, colon	(299)
CD45^+^RORγt^+^	CD3ε, CD19	SI LP	(300)
CD45^+^RORγt^+^	CD3ε, CD11b, B220, Ly6G/6C	SI LP	(301)
CD45^+^RORγt^+^	CD3ε, CD5, CD19	SI LP, colon LP, mLN	(302)
CD45^+^RORγt^+^	CD19, CD3	SI LP	(303)
CD45^+^RORγt^+^	CD3, CD4, CD8, CD19, Ly6G/6C, Ter119	SI LP	(304)
CD45^+^RORγt^+^	CD3, CD11b, B220, Ly6G, Ter119	Colon LP	(190)
CD45^+^RORγt^+^	CD3, Ly6G/6C, CD11b, B220, Ter119	Colon, mLN	(305)
CD45^+^RORγt^+^	CD3ε, CD11b, B220, Ter119, Ly6G/6C	Colon LP	(306)
CD45^+^RORγt^+^	CD3	Lung	(307)
CD45^+^RORγt^+^	CD3ε, NK1.1, Ly6G/6C, CD11b, B220, Ter119	Cornea, conjunctiva, lacrimal gland	(308)
CD45^+^RORγt^+^KLRG1^−^	CD3, CD5, B220, CD11c, NK1.1	SI, Colon, cecum LP	(242)
CD90^+^	CD3e, CD8α, B220, CD11b, CD11c	Neonatal SI LP	(309)
CD90^+^CD117^+^KLRG1^−^	CD3ε, CD8α, CD11b, CD11c, CD19, B220, Ly6G/6C, TCRβ, TCRγ/δ, Ter119, NK1.1	Spleen, SI	(310)
CD90^+^CD127^+^	CD3, CD11c, B220	Spleen	(311)
CD90^+^CD127^+^CD117^+^	CD3, B220, CD11c, Ly6G/6C, NK1.1	Spleen	(312)
CD90^+^CD127^+^CD25^+^	CD3, CD19, CD11c, NK1.1	mLN	(313)
CD90^+^CD127^+^KLRG1^−^	CD3, CD11b, CD11c, CD14, CD19, TCRβ, TCRγ, NK1.1	SI LP, colon LP	(314)
CD90^+^CD127^+^KLRG1^−^Sca-1^lo^	CD3ε, CD4, CD8α, CD5, NK1.1, B220, CD11b, CD11c, Gr-1, FcεRIα, Ter119	SI LP	(315)
CD90^+^CD127^+^RORγt^+^	CD5, CD3, CD11b, CD11c, B220, Ly6G/6C, Ter119	Colon	(316)
CD90^+^CD127^+^RORγt^+^	CD3, CD5, B220, NK1.1, F4/80, Ly6G/6C	Colon LP	(317)
CD90^+^CD127^+^RORγt^+^	CD3, Ly6C/6G, CD11b, B220, Ter119	SI	(318)
CD90^+^CD127^+^RORγt^+^	CD3, CD11b, Ly6G/6C, B220, NK1.1, CD11c, Ter119	Lung	(319)
CD90^+^CD127^+^RORγt^+^	Data not found	Cecum, mLN	(320)
CD90^+^CD127^+^RORγt^+^	CD3, CD5, B220, CD11b, Ly6G/6C, Ly6B, Ter119	Lung	(321)
CD90^+^CD127^+^RORγt^+^	CD3ε, TCRβ, GL3, CD19, Ly6G/6C, CD11b, F4/80, Ter119, NK1.1, CD49b, CD11c	LN, spleen	(322)
CD90^+^CD127^+^RORγt^+^	CD3ε, CD11b, B220, Ter119, Ly6G/6C	SI LP	(323)
CD90^+^CD127^+^RORγt^+^KLRG1^−^	CD3, CD5, CD19, CD11b, TCRγδ	Colon LP, SI LP	(37)
CD90^+^IL-23R^+^	CD3	SI LP	(324)
CD90^+^RORγt^+^	CD3, CD8α, TCRβ, TCRγδ, CD11b, CD11c, B220, Ly6G/6C, NK1.1, Ter119	SI	(325)
CD90^+^RORγt^+^	CD3, TCRγδ, CD11b, NK1.1	Colon LP	(33)
CD90^+^RORγt^+^	TCRβ, TCRγδ, CD19, Ly6G/6C, Ter119, NK1.1, CD11c, CD11b	Colon LP	(326)
CD90^+^RORγt^+^	Data not found	Colon LP	(327)
CD90^+^RORγt^+^	CD5, CD8, CD3, B220, CD11c, CD11b, T-bet	SI	(328)
CD90^+^RORγt^+^	CD3	Colon LP, SI LP	(329)
CD90^+^RORγt^+^KLRG1^−^	CD3, CD8, CD11c, CD19, B220, Ly6G/6C, TCRβ, TCRγδ, Ter119	SI, colon	(330)
RORγt^+^	CD3ε, CD5, CD8α, CD19, Ter119, Ly6G/6C, TCRβ, TCRδ	Spleen, bone marrow, SI LP	(331)
RORγt^+^	CD3, CD19, B220, CD11b, CD11c, Ter119	SI LP	(332)
RORγt^+^	CD3e, CD11b, B220, Ter119, Ly6G/6C	PP	(228)
RORγt^+^	CD3	Colon LP	(333)
RORγt^+^	CD3, CD4, CD8, CD16, CD19, CD11c, FcεR1α	Colon LP	(334)
RORγt^+^	CD3ε	SI LP	(335)
RORγt^+^	CD3ε, CD5, CD19	SI LP	(231)
RORγt^+^	CD3, CD19	SI LP I IE	(336)
RORγt^+^	CD3, CD5, CD19, B220, Ly6G, CD11b, CD11c, Ter119	SI LP, colon LP	(229)
RORγt^+^	CD3, B220, CD11b, CD11c	Spleen, colon LP, SI LP	(337)
RORγt^+^	CD3, B220, CD11b, CD11c	Colon LP	(338)
RORγt^+^	CD3ε	SI LP	(339)
RORγt^+^	CD11b, CD11c, Ter119, B220, CD3ε, Ly6G/6C, TCRβ	SI LP	(340)
RORγt^+^	CD3ε, B220, CD11b, CD11c	SI, colon	(341)
RORγt^+^CD161^+^KLRG1^−^	CD3, TCRβ, CD11b, CD14, CD19, B220, TCRγδ, NK1.1	SI LP	(342)
RORγt^+^IL-22^+^	Without	SI LP	(343)
RORγt^+^MHC II^+^	Without	SI LP	(344)
RORγt^+^MHC II^+^	CD3, CD19, NK1.1, CD11b, B220, F4/80, CD11c, Ly6G/6C, FcεR1α	Spleen, mLN	(345)

LP, lamina propria; PL, peritoneal lavage; PP, Peyer’s patches; SI, small intestine; LN, lymph node; mLN, mesenteric lymph node.

**Table 3:** Markers used for the identification of human and mouse ILC3.

Human				Mouse			
Markers	*n*	Combinations	*n*	Markers	*n*	Combinations	*n*
CD127	114	CD127^+^CD117^+^CD294^−^	48	RORγt	189	CD127^+^RORγt^+^	60
CD117	112	CD127^+^CD117^+^CD161^+^CD294^−^	25	CD127	117	RORγt^+^	48
CD294	79	CD127^+^CD117^+^	24	CD90	89	CD90^+^RORγt^+^	32
CD161	30	CD127^+^CD117^+^CD161^+^	5	KLRG1	19	CD90^+^CD127^+^RORγt^+^	20
RORγt	7	CD127^+^	5	CD117	16	CD90	10
IL-23R	1	CD117^+^CD294^−^	4	IL-17	4	CD127^+^CD117^+^RORγt^+^	8
		CD127^+^RORγt^+^	4	IL-23R	3	CD90^+^RORγt^+^KLRG1^−^	5
		CD117^+^	4			CD90^+^CD127^+^	5
		CD117^+^RORγt^+^	2			CD127^+^	5
		RORγt^+^	2			CD127^+^CD117^+^	4
		CD127^+^IL-23R^+^	1			CD90^+^CD127^+^RORγt^+^KLRG1^−^	4
		CD127^+^CD294^−^	1			CD117^+^RORγt^+^	3
						Rest	30

n, number of studies.

## 3 Lineage cocktails

CD3 is a marker that is targeted by almost all lineage cocktails. Additionally, in some of the mouse studies, CD3 is the only negative marker used for ILC3 identification. The logic behind this strategy is clear when RORγt is used as the key positive ILC3 marker. As RORγt is expressed by T cells and ILC3 (346), the latter can be identified as CD3^−^RORγt^+^ cells. It is known that human bone marrow and blood neutrophils, as well as mouse lung neutrophils, can express RORγt (347, 348). However, neutrophils are easily distinguished from the lymphocytic populations by flow cytometry using forward and side scatter. Also, there is a report on a RORγt-expressing DC subset in the spleen (349), while the existence of a similar subset was not confirmed in the intestine (98). Thus, CD3 and RORγt can be used as the basic discriminators of ILC3 in the murine intestine, but not the spleen. As for human samples, it seems that RORγt can be expressed by all ILC subsets, as well as by NK cells (24), thus making it an unsuitable marker for ILC3 discrimination.

Other T-cell markers can be used instead of CD3, such as TCRαβ and TCRγδ. Lineage cocktails used in some studies contained both CD3- and TCR-specific antibodies, even though this combination seems redundant having in mind that CD3 already defines all TCR-positive cells. Additionally, CD4 and CD8a were used as lineage markers in some studies. While there are no data on the expression of CD8 in ILC3, there are studies showing that the LTi ILC3 subset can express CD4 (160, 350), thus making CD4 inadequate as a lineage marker. Also, there are data on the expression of RORγt by CD3^+^CD4^−^CD8^−^ cells in patients with systemic lupus erythematosus and psoriasis (351, 352), which contribute to the view that CD3, and not CD4 and CD8, should be used in lineage cocktails. CD5-specific antibodies are also frequently used in mice, as this marker is specific for T cells and a subset of B cells.

The second most commonly used lineage markers are B-cell markers, including B220 (CD45RA) and CD19. CD45RA is an isoform of CD45, a molecule expressed on all types of hematopoietic cells except mature erythrocytes and platelets (353). Although it is expressed in mouse DC (354), naive T cells (355), and T cells undergoing apoptosis (356, 357), it is considered a pan-B-cell marker in mice, present on pre-B cells and at all stages of B-cell maturation (358), making it a suitable negative marker for phenotyping ILC. CD19 is a molecule belonging to the immunoglobulin superfamily, and its expression is specific to normal and neoplastic B cells, as well as follicular DC (359). Both B220 and CD19 molecules are suitable B-cell markers in mice, whereas in humans, B220 is detected on ILC3 in the blood and cord blood, with heterogeneous expression in tonsillar, intestinal, and lung ILC3 (360). Therefore, CD19, and not B220, should be used as a B-cell-related lineage component for human ILC detection.

The most challenging distinction is the one between NK and ILC3 cells. NK cells and ILC share the same progenitor, while the mature forms of ILC also share some markers with NK cells. For instance, ILC1, some ILC3, and NK cells express T-bet. Therefore, it is important to distinguish these cells when detecting ILC3. NK1.1-specific antibodies are frequently used in mice lineage cocktails, while CD94 (Klrd1) is preferentially used in human samples to exclude NK cells, although the use of NKG2A has also been reported in some studies. Additionally, CD56 is used to exclude NK cells and other immune cells able to express this molecule, such as T cells, DC, and monocytes (361). Some researchers also use markers specific for lymphocyte subpopulations, such as CD49b for some T cells (Tr1, NKT), Klrg1 for effector memory CD8^+^ T and NK cells, or CD27 for activated effector T cells, NK cells, and activated B cells. One important issue is that different mouse strains carry different NK-related markers. For example, the Balb/c strain lacks NK1.1 (362), so CD49b can be used for the determination of NK cells.

CD11b, CD11c, Gr1, Ly6G, Ly6C, and Ly6B are markers that are used in lineage cocktails to eliminate monocytes, DC, granulocytes, macrophages, NK cells, and subsets of T and B cells. Additionally, F4/80 is a relevant marker of murine macrophages. FcϵRIα is also used to detect epidermal Langerhans cells, eosinophils, mast cells, basophils, and various antigen-presenting cells. However, there are reports on ILC3 expressing CD11b and CD11c (27), thus compromising their use in lineage cocktails. Finally, Ter119 is routinely used to exclude erythrocytes from analyses.

Different lineage cocktails can be used while studying ILC3. It is on the researchers to make the best choice for a particular species and tissue in which ILC3s are being detected. It seems that the uncritical use of redundant markers in the lineage cocktails does not improve the efficacy of ILC3 detection, while the use of markers that are potentially expressed by ILC3 increases the risk of excluding some ILC3 subpopulations from the analysis. So, the advice on lineage cocktails might be the following: keep it simple and efficient.

## 4 Markers used for the discrimination of ILC3

Various markers are used, alone or in combination, to identify ILC3. CD45 is a marker of hematopoietic cells and is, therefore, expressed by ILC3. The use of anti-CD45 antibodies is essential when identifying ILC3 in the intestine, lungs, and other organs and tissues where there is plenty of non-hematopoietic cells. There, gating on CD45^+^ cells as one of the first steps in flow cytometry analysis is a prerequisite to prevent contamination with non-hematopoietic cells and to get precise data on ILC3. On the other hand, identification of ILC3 in the peripheral blood, spleen, tonsils, or lymph nodes does not require the use of anti-CD45 antibodies, as there are few non-hematopoietic cells, if any. Still, it is clear from **Tables 1**, **2** that some authors do not use CD45-specific antibodies where necessary, and the results of such studies should be carefully considered.

**Table 4** presents the most common markers used in ILC3-related studies. Both CD nomenclature and alternative names are used in research papers, thus confusing readers who are not specialized in the ILC3 field. While alternative names have the advantage of functional comprehension and historical connotation, CD designation offers a more systematic approach. Thus, it seems productive to use CD nomenclature as much as possible, or at least to provide information on CD designation in the papers that use alternative names.

**Table 4:** Cluster of differentiation (CD) and alternative nomenclature of common ILC3 markers.

CD nomenclature	Alternative names	Function
CD90	Thy-1	Not elucidated, cell adhesion
CD117	c-kit, SCFR	Receptor tyrosine kinase
CD127	IL-7Rα	IL-7 receptor
CD161	Klrb1b, NKRP1A	Negative regulator of cytotoxicity
CD196	CCR6	Chemokine receptor for CXCL20
CD294	CRTH2, DP-2	Prostaglandin D2 receptor 2
CD335	NCR1, NKp46	Cytotoxicity triggering receptor
CD336	NCR2, NKp44	Cytotoxicity triggering receptor

CCR6, C-C motif chemokine receptor 6; CRTH2, chemoattractant receptor-homologous molecule expressed on TH2 cells; IL-7R-α, interleukin-7 receptor subunit alpha; Klrb1b, killer cell lectin-like receptor subfamily B, member 1; Klrd1, killer cell lectin-like receptor subfamily D, member 1; NCR, natural cytotoxicity triggering receptor; SCFR, mast/stem cell growth factor receptor; Thy-1, thymocyte differentiation antigen 1.

### 4.1 CD127

According to the literature (**Figure 1**; **Table 3**), CD127 is the most commonly used ILC3 marker in humans and the second most common in mice. This marker is an α subunit of the IL-7 receptor. IL-7 is important for the early development of T and B cells from bone marrow precursors and for the development of T cells in the thymus (363). As IL-7 is the major growth factor of ILC, but not NK cells, CD127 is expressed by the former (364), but not by the latter, and is thus a valuable marker for the distinction of these two cell types. In addition to ILC, CD127 is expressed in naive (365), memory (366), and regulatory T cells (367). Therefore, using T-cell-specific antibodies in the lineage cocktail is necessary to be able to use CD127 as a reliable marker of ILC. This marker is of particular interest for discriminating between NK and ILC1 cells, as both cell types are known to express T-bet. Still, keeping in mind that NK cells do not express RORγt, CD127 does not appear to be essential for the discrimination between ILC3 and NK cells. It seems that it is reasonable to use CD127 in ILC3 research when analyzing all ILC subsets, while it is not essential for ILC3 subset identification.

### 4.2 CD117

This marker is used in ILC3 studies in humans almost as frequently as CD127 is, yet its use in mouse studies is not so frequent (**Figure 1**; **Table 3**). CD117 is a tyrosine kinase, also known as c-Kit, and it is the receptor for the SCF. CD117 plays an important role in early hematopoiesis and its expression is lost during cellular differentiation (368). However, in addition to mature mast cells and melanocytes, CD117 is also expressed on recently activated human CD8^+^ T cells (369) and circulating B cells (370). Within the ILC, CD117 is expressed on ILC2 and ILC3 (17). Since CD117 is not exclusively expressed on ILC3, additional markers should be used to distinguish between ILC2 and ILC3, and its use may not be crucial for detecting ILC3.

### 4.3 CD294

This marker is frequently used in human but not in mouse studies. CD294, also known as CRTH2, is a prostaglandin D2 receptor expressed on Th2 cells and ILC2 in humans, but not in mice. It is also present in both human and mouse eosinophils and basophils (371), while it is not expressed on ILC3 (17). Thus, when phenotyping ILC3, it serves to exclude the ILC2 population. CD294 is most often used in combination with CD127 and CD117, as based on the expression of these three markers in lineage-negative cells, all three ILC subsets can be distinguished. However, when only detecting the ILC3 population, it does not seem necessary to use all of these markers.

### 4.4 CD90

This marker is very frequently used in mouse but not in human studies, although the mouse and human CD90 proteins are highly similar. Despite this, CD90 expression is negligible in human cells and, thus, cannot be used as a marker for ILC (372). CD90 is a glycophosphatidylinositol-anchored cell surface protein and a member of the immunoglobulin superfamily. While its function is still not fully elucidated, it has been shown to interact with integrins and is therefore presumably important for cell adhesion (274). Within immune cells, CD90 is expressed in T cells, NK cells, and ILCs. It is found on the surface of all subtypes of ILC (373). In Rag1^−/−^ mice, the anti-CD90 antibody is commonly used to deplete ILC (374). Although CD90 is considered a pan-ILC marker, a recent study showed that CD90 is not constitutively expressed in intestinal ILC (375). Additionally, having in mind that CD90 is expressed in all ILC subsets, CD90 does not appear to be the best choice for the detection of ILC3. Furthermore, based on their results of different expressions of CD90 on ILC, Schroeder et al. (375) suggested that CD127 is a much more reliable ILC marker than CD90.

### 4.5 CD161

CD161 is used as an additional marker to determine ILC3 in humans, but not in mice. CD161 is a human homolog of NK1.1 and is expressed in NK cells, T cells, NKT cells, monocytes, and DCs (376). It is used in combination with CD127 and CD117, with or without CD294 (**Figure 1**; **Table 3**). Aside from ILC3, CD161 is also expressed on ILC2 (377). Therefore, this molecule cannot be used as a basic, but only as a supplementary marker for ILC3 phenotyping.

### 4.6 RORγt

RORγt is by far the most frequently used marker for the discrimination of ILC3 in mouse studies, but not in humans. This molecule is expressed by T cells and ILC3 (346). Human bone marrow and blood neutrophils, as well as mouse lung neutrophils and splenic DCs, can also express RORγt, as previously mentioned (347–349). However, in human secondary lymphoid tissues, including the tonsils, lymph nodes, and spleen, RORγt is expressed in all ILC subsets, as well as in NK cells. In these tissues, a novel Lin^−^CD34^+^CD45RA^+^CD117^+^IL-1R1^+^RORγt^+^ cell population was found, capable of differentiating into all major ILC populations (24). The expression of RORγt was also found in human fetal gut and adult peripheral blood ILC2 cells (377). Although there are no data showing the expression of RORγt in adult human intestinal ILC1 and ILC2, Cogswell et al. (378) have demonstrated the expression of RORγt in ILC1, ILC3, and NK cells in the colon of rhesus macaques, which additionally compromises the use of RORγt in the identification of ILC3 in humans. However, in all of the abovementioned studies, the expression of RORγt was much higher in ILC3 than in other ILC subsets. Thus, RORγt is the crucial marker for the identification of ILC3 in mice and a supplementary one in humans.

## 5 Molecules important for the identification of ILC3 subsets

### 5.1 CD196

CD196 or CCR6 is a G protein-coupled receptor expressed on DCs, CD4^+^ T cells, B cells, and ILCs. CCL20 is a high-affinity ligand for CCR6, and pro-inflammatory cytokines have been shown to induce its expression (379). CCL20 is highly present in inflamed tissues and drives the recruitment of CCR6-expressing immune cells (380). Within ILC3, CCR6 is expressed on LTi and LTi-like cells (381) and is an important marker for identifying these cells. While CCR6-expressing ILC3s are important for the formation of lymphoid organs during development, in adult mice, these cells are usually aggregated with DCs, B cells, and stromal cells in cryptopatches and isolated lymphoid follicles (382). Thus, CD196 is a valuable marker for the identification of ILC3 subpopulations.

### 5.2 CD335

CD335 or NKp46 is a member of the immunoglobulin superfamily and one of the NCRs of NK cells. While there are three NCRs in humans, namely, NKp30, NKp44, and NKp46, the only NCR described in mice is NKp46 (383). It is expressed on the surface of non-activated and activated NK cells (384), a small population of T lymphocytes, ILC1, and a subset of ILC3, called NCR^+^ ILC3 (385). Although the functional role of NKp46 on ILC3 is poorly understood, this molecule is essential for the identification of ILC3 subsets.

### 5.3 CD336

CD336 or NKp44 is another NCR and is expressed only in humans. Like NKp46, it belongs to the immunoglobulin superfamily, but there is no homology between these two molecules (386). While NKp30 and NKp46 are constitutively expressed in human NK cells, NKp44 is expressed only upon activation (384), and the use of anti-NKp44 antibodies has been shown to stimulate cytokine production by human ILC3 (387). In addition to NK cells, NKp44 is also expressed on ILC1 and ILC3 (388) and is thus helpful in defining ILC3 subsets.

## 6 ILC3 subsets in humans

**Figure 2:**
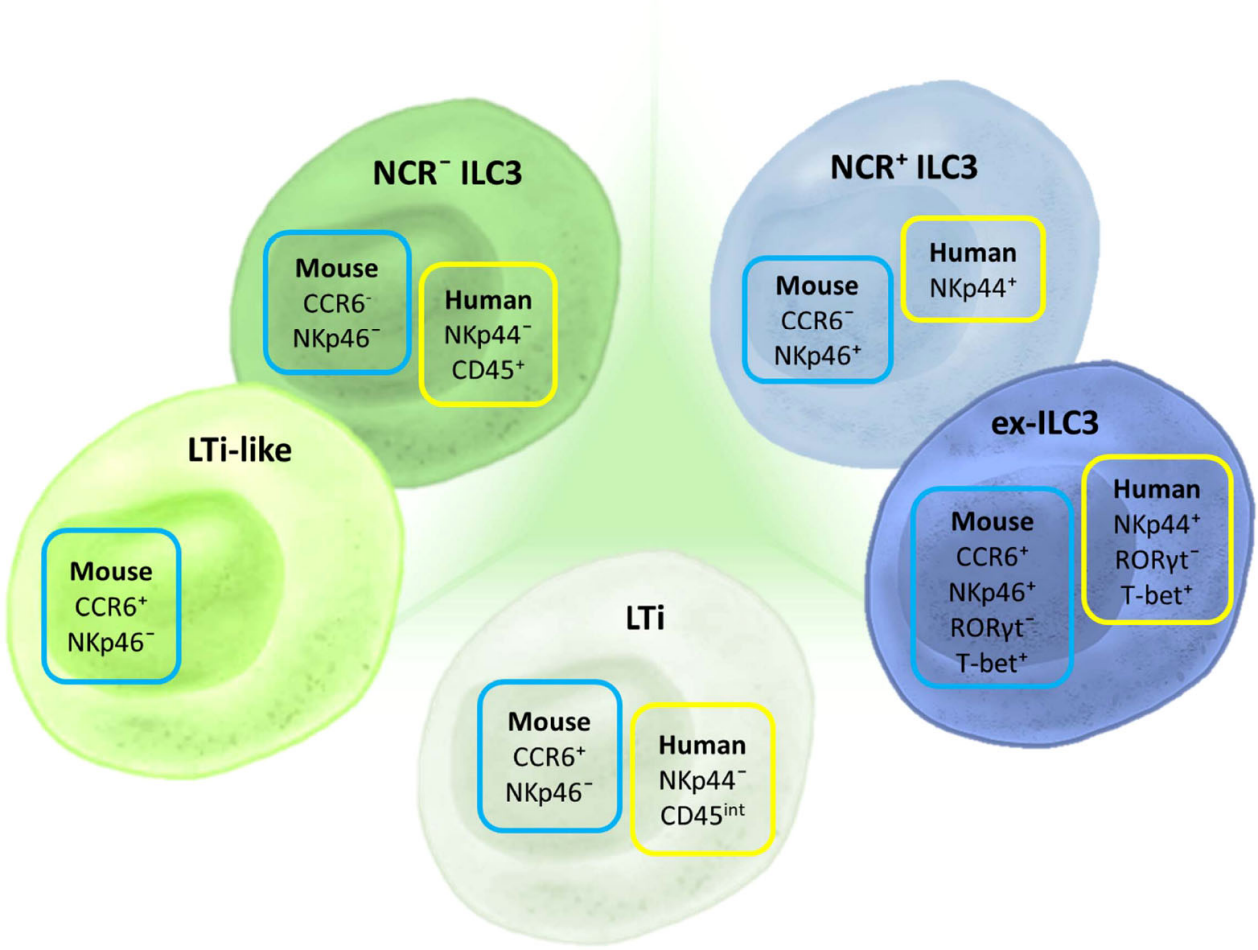
Markers for ILC3 subset discrimination in humans and mice. The basic classification of ILC3 includes three subtypes in humans and mice: LTi, NCR^+^, and NCR^−^ ILC3. In mice, an additional subpopulation can be identified—LTi-like cells, although it is still not clear if this subpopulation is distinct from NCR^−^ ILC3. Different NCRs are used for characterizing human and mouse ILC3s: NKp44 and NKp46, respectively. CCR6 is an additional marker used for ILC3 classification in mice. Aside from these basic subtypes, there is a defined group of ILCs that shares certain markers and properties of both ILC3 and ILC1. These cells are considered to have converted from ILC3 toward ILC1 and are thus termed “ex-ILC3.” They are distinguished from NCR^+^ ILC3 by the expression of RORγt and T-bet.

Three different ILC3 subtypes can be distinguished in humans (**Figure 2**). LTi cells are the subtype that is most distinct by their developmental pathway and functional properties, and some even consider them to be a separate cell population (385). However, when it comes to their identification by flow cytometry, there is a clear lack of appropriate markers (385). Two other important subtypes are distinguished in humans based on the expression of CD336 (NKp44) and are referred to as NKp44^−^ ILC3 and NKp44^+^ ILC3 (38, 48, 387, 389). Still, LTi cells are also defined as NKp44^−^. The distinction between NKp44^−^ ILC3 and LTi in humans can only be performed through analysis of CD45 expression. While NKp44^−^ ILC3s have high CD45 expression, LTi cells express intermediate levels of CD45 (385). Thus, we are in need of a novel marker that would make a clear distinction between NKp44^−^ ILC3 and LTi. An additional subpopulation of NKp44^+^ ILC3 that loses the ability to express RORγt but keeps the expression of T-bet, and thus becomes closer to the ILC1 phenotype, can be identified as “ex-ILC3” cells (390).

ILC3s in humans are most often found in mucosal tissue and are most abundant in the intestine, where they exhibit several functions (30). For instance, it was shown that after gastrointestinal transplantation, the recipients’ ILC rapidly infiltrated the graft (95). Increased frequencies of human NKp44^+^ ILC3 were observed in the graft, which has been associated with successful intestinal transplants as it reduces the risk of rejection (99). A similar observation was made in leukemic patients after hematopoietic stem cell transplantation (95). These observations have further strengthened the hypothesis that NKp44^+^ ILC3s are essential for maintaining homeostasis and protecting the gastrointestinal tract. However, there are conflicting data from some cancer studies that the role of IL-22-producing NKp44^+^ ILC3 may be detrimental to patients. For example, patients with hepatocarcinoma had more NKp44^+^ ILC3s than healthy controls (48). On the other hand, NKp44^–^ ILC3s have been described to exacerbate inflammation, especially in inflammatory bowel disease, due to their secretion of IFN-γ and IL-17A (122, 390, 391). Additionally, it was shown that upon activation, human ILC3s were able to induce antigen-specific CD4^+^ memory T-cell responses (392). Both NKp44^+^ ILC3 and NKp44^–^ cells can produce IL-17, but the ability to produce IL-22 is mainly restricted to NKp44^+^ ILC3 (387). By producing IL-22, NKp44^+^ ILC3s promote tissue integrity, maintain barrier functions, and promote homeostasis (238), particularly in the gastrointestinal tract. IL-22 secretion by NKp44^+^ ILC3 is activated after various stimuli such as food intake (241, 314), danger signals (111, 393), and changes in the cytokine milieu (6, 7). In addition, NKp44^+^ ILC3 can interact with other cells through the expression of neuroregulatory receptors which allows them to directly interact with glial cells (246) and goblet cells (394).

The attempt to classify ILC3 subgroups becomes more complex when different tissues are observed. For example, single-cell RNA sequencing analysis has shown that three populations can be identified in the tonsils based on the expression of NKp44, human leukocyte antigen D-related (HLA-DR), and CD62L, and each of these subpopulations has a different cytokine profile (395). HLA-DR^+^ ILC3s have also been observed in the human intestine (138, 396). Apart from this, NKp44^+^ ILC3s can be further divided into two populations based on the expression of neuropilin-1 (NRP-1). NRP-1^+^ ILC3s produce more cytokines than NRP-1^−^ ILC3, and these cells have been detected only in lymphoid tissues and inflamed lung tissue (105). Overall, these data make it necessary to find a consensus in identifying and classifying ILC3s into specific subtypes.

It was generally presumed that all human ILC3s express a common transcription factor RORγt, which implied that RORγt is essential for their functions. However, in patients with RORγt deficiency, IL-22^+^ but not IL-17^+^ ILC3s were found. Similarly, *in-vitro* experiments showed that chemical inhibition of RORγt did not affect the ability of ILC3 to produce IL-22 (40). This suggests that other transcription factors such as AhR or GATA3 may regulate IL-22^+^ ILC3, as studies in mice have found that these transcription factors are implicated in the regulation of IL-22 production by ILC3 (161, 397).

## 7 ILC3 subsets in mice

Discrimination among ILC3 subgroups in mice is mainly based on the expression of two cell markers: CD196 (CCR6) and CD335 (NKp46) (**Figure 2**). There are three basic populations of murine ILC3: NCR^+^ ILC3 (CCR6^−^NKp46^+^), NCR^−^ ILC3 (CCR6^−^NKp46^+^), and LTi cells (CCR6^+^NKp46^−^). LTi cells identified postnatally are referred to as LTi-like. Additionally, there is a subpopulation of NCR^+^ ILC3 that is called “ex-ILC3,” which is characterized by diminished RORγt and upregulated T-bet expression.

LTi cells are vital for the formation of secondary lymphoid organs throughout the prenatal period as they produce lymphotoxin-α1β2, which activates lymphoid tissue organizer cells in future lymph node and Peyer’s patch (PP) sites (15, 398). Post-birth, LTi-like cells also play a role in the development of cryptopatches, small lymphoid aggregates in the gut, which can grow into isolated lymphoid follicles (ILFs) in response to microbial signals (398). CCL20, the major CCR6 ligand, is constitutively expressed within the intestinal PP, ILF, and mesenteric lymph nodes (mLNs), confirming the importance of CCR6-mediated signals for secondary lymphoid organogenesis. These signals also help keep leukocytes at sites critical for immune surveillance (399). Accordingly, CCR6^+^ LTi-like ILC3s are the prevailing ILC3 subset in the PP, mLN, and colon lamina propria (243). In the intestine, LTi-like cells are also important for maintaining local gut homeostasis, through their interactions with both the innate and adaptive immune responses. For instance, LTi-like cells can produce IL-22 and IL-17, which are able to increase the expression of intestinal antimicrobial peptides (350, 400).

Additionally, LTi-like cells, unlike CCR6^−^ ILC3, constitutively express MHC II in the gut while lacking the expression of co-stimulatory molecules CD40, CD80, and CD86, compared with DCs (369). This is one of the features that allows them to play a part in maintaining tolerance through the suppression of commensal antigen-specific T effector cells, enhanced generation of memory T cells, and promotion of mucosal antibody responses [reviewed by Zhong et al. (14)]. LTi-like cells can also be separated into CD4^+^ and CD4^−^ subsets, even though gene expression analysis showed that there were minimal differences between the two groups—probably not enough to allow functional significance (288). Aside from confirming that CCR6 transcripts were expressed at higher levels in LTi-like cells compared with other ILC3s, the same was shown for an additional chemokine receptor, CXCR5 (288). Finally, fate-mapping studies have demonstrated that while the LTi lineages develop from ILC progenitors that have never expressed the transcription factor PLZF, all other ILC3 subtypes are generated from progenitors that do express it (401). GATA3 is another transcription factor that was shown to be necessary for the development of non-LTi ILCs, while it was not required for the generation of LTi-like cells (402). Additional precursor single-cell analyses (403) and the use of multitranscription factor reporter mice (404) confirmed the distinct origins of ILC and LTi lineages. Based on the differences in gene expression needed for their development, LTi-like cells could be considered a separate group of ILCs, despite sharing similarities in phenotype and function with NKp46^+^ ILC3.

NKp46^+^CCR6^−^ ILC3, unlike CCR6^+^ LTi-like cells, are marked by T-bet expression. Their postnatal development from NKp46^−^CCR6^−^RORγt^+^ progenitors, often labeled as double negative (DN) ILC3, is in part guided by commensal microbiota signals and IL-23, while c-Maf-controlled graded T-bet expression ensures the expression of NKp46 (247, 405). To maintain their phenotype, NKp46^+^ ILC3s also require Notch signaling, which can be regulated by both AhR (406) and T-bet (407), while it was shown that TGF-β impairs their development (290).

In the gut, NKp46^+^ ILC3s are predominantly found in the small intestine lamina propria (243), and their localization within the lamina propria villi is influenced by CXCR6 (160). NKp46^+^ ILC3s also make up most of the ILC3s in the cecum and are necessary for its homeostasis (289). NKp46 has mostly been studied as one of the NCRs that are expressed by NK cells and whose activation by various pathogen-derived ligands can trigger NK cell-mediated cytotoxicity accomplished by the secretion of perforin or proinflammatory cytokines such as IFN-γ and TNF or through activation-induced cell death. Unlike NK cells, it seems that intestinal NKp46^+^ ILC3s are not able to perform these functions, while they do produce IL-22 to fight intestinal inflammation (3), similar to LTi-like cells. Interestingly, questions about the redundancy of the NKp46^+^ subgroup of ILC3s have been brought up in some experimental settings. For instance, NKp46^+^ ILC3s were shown to be inessential in the fight with a *Citrobacter rodentium* infection, when compared with LTi-like ILC3s (256). More specifically, IL-22 was necessary for the resistance to *C. rodentium*, but IL-22 production by NKp46^+^ ILC3 was not (289). However, NKp46^+^ ILC3s also have the potential to secrete GM-CSF and were crucial in promoting the accumulation and activation of inflammatory monocytes in an anti-CD40-induced colitis model (256).

A fate-mapping study by Viant et al. (290) demonstrated a notable proportion of NKp46^−^ ILC3 used to express NKp46, and this phenomenon was predominantly observed in mucosal tissues but not in the bone marrow. Furthermore, an RORγt fate-mapping study revealed that, in some inflammatory conditions, NKp46^+^ ILC3 can significantly lower or lose RORγt expression, while T-bet expression increases. At the same time, these “ex-ILC3s” start producing IFN-γ and upregulate perforin and granzyme B expression, becoming more akin to ILC1 (9). Following *Salmonella typhimurium* infection, these cells were the main source of IFN-γ in the small intestine (405), which implies an adaptive role in microbial defense. On the other hand, NKp46^+^ ILC3 plasticity is regulated by c-Maf, which suppresses their conversion into the ILC1-like state by restraining T-bet expression and supporting RORγt activity (219, 247). Finally, the transcriptional profile of NKp46^+^ ILC3 was shown to have features in between those of NKp46^−^ LTi-like ILC3 and NKp46^+^ ILC1 (while sharing more transcripts with NKp46^+^ ILC1). This might be the basis of their plasticity potential, which is probably shaped by their specific microenvironment (288).

## 8 ILC3 sorting and culture

Our literature survey shows that a consensus about the minimal or optimal panel of surface markers for ILC3 sorting is still missing. However, in most sorting protocols, the live/dead discrimination and doublet exclusion seem to be mandatory. Some authors suggest that before cell sorting, enrichment of CD90^+^ (233) or Lin^−^ cells (408) should take place. The most efficient sorting of mouse ILC3 occurs in samples obtained from mice that carry a fluorescently labeled reporter RORγt. In this case, the sorting protocol might include only a CD3 staining, after which ILC3s are sorted as CD3^−^RORγt^+^ cells (the exclusion of Th17 cells is achieved). However, most of the protocols include additional surface markers—ILC3 can be sorted as CD45^int^CD90^high^RORγt^+^ (275) or as Lin^−^CD45^+^CD90.2^+^RORγt^+^ (270). In the studies not using RORγt reporter mice, investigators tend to use as many markers as they can for the determination of ILC3. For example, Zhou et al. (96) sorted an enriched population of ILC3 as Lin^−^CD45^low^CD90.2^high^CD127^+^KLRG1^−^. However, other groups used fewer denominators for the enrichment of ILC3, for example, the CD3^−^ and CD90^high^CD45^low^ gating (254) or complete lineage-negative population coupled with CD90^high^ and CD45^low^ markers (238, 269). Some groups use KLRG1 as an additional discriminative factor for ILC3 enrichment in the following panel Lin^−^CD90.2^+^KLRG1^−^ (330) or Lin^−^CD45^+^CD90.2^+^KLRG1^−^ (128). As recent data indicate that surface CD90 can be downregulated when ILC3s are functionally active, the usage of CD90 for ILC3 sorting is questionable (375). CD127 and ST2 are also used to determine ILC3 for sorting in addition to Lin^−^CD45^+^CD4^−^. The same study uses the NKp46 marker to sort-purify NCR^+^ and NCR^−^ ILC3s (122). In all articles that we have analyzed so far, the percentage of acquired RORγt^+^ cells after ILC3 sorting (without the use of knock-in fluorescent labeling of RORγt) was not determined. Therefore, cells sorted in such a way can be referred to as enriched ILC3s, or just ILCs (as they are composed out of all three ILC types). The recommendation for future studies where these cells are used *in vitro* would be to evaluate the ILC distribution in post-sorted samples before engaging in any treatment or stimulation.

Human ILC3 cell sorting tends to be more difficult, as their surface markers may be different in peripheral blood mononuclear cells (PBMCs) and within the tissues. Also, unlike in mice, human ILC2 and ILC3 cannot exclusively be defined by transcription factors GATA3 and RORγt (409); therefore, there is no need for confirmation in post-sort samples. In general, ILC3 sorting from the PBMC or colon was performed according to the surface markers Lin^−^CD45^+^CD127^+^CRTH2^−^CD117^+^ (410, 411). However, when ILC3s are sorted from the lungs, additional markers were included such as CD16^−^NKG2A^−^ and CD161^+^ (411).

ILC3 sorting is usually done in order to perform single-cell RNA sequencing, *in-vitro* culture, or cell transfer. *In-vitro* culture of enriched human ILC3 is a prolonged process that requires several growth factors (SCF, IL-7, Flt-3L, IL-2, IL-15). The success of ILC3 propagation *in vitro* can depend upon a layer of cells to support ILC3 proliferation (such as mesenchymal stem cells) (79). Mouse-enriched ILC3s are usually cultured with SCF and IL-7 and on occasion with the addition of IL-1β, IL-12, or IL-23. *In-vitro* culture and stimulation of ILC3 with PMA/ionomycin/brefeldin is done in order to detect ILC3-derived cytokines. However, this can lead to the downregulation of ILC3-specific surface markers, thus interfering with flow cytometry analysis of post-treatment ILC3 (412). For performing a cell transfer, ILC3s are usually used freshly sorted from cell suspension derived from intestinal lamina propria or the lungs. Lamina propria is most often chosen as the source of ILC3 because of the abundance of ILC3s in the innate immune cell compartment. As the numbers of obtained ILC3 from tissues are very low, researchers use from 100,000 to 500,000 cells per transfer (233, 267). Having in mind the required precision in ILC3 phenotyping, sorting, and culture conditions and the paucity of tissue ILC3, as well as the limited availability of organs/tissues for their isolation, the use of ILC3 for human cell therapy seems to be complex and unrealistic. Still, alternative strategies, such as propagating tissue-like ILC3 from CD34^+^ hematopoietic progenitors (79), could overcome the above-stated obstacles on the road toward ILC3-based therapy.

## 9 Conclusions

The ILC3 research field is still relatively novel, and ILC3 identification by flow cytometry is not yet standardized. The expanding complexity of phenotypic determinants of ILC3 and the tissue specificities of ILC3 contribute to the discrepancies in protocols used by different research groups. Through analysis of the current state of phenotypic characterization of ILC3 by flow cytometry, we conclude that markers for the identification of ILC3 should be chosen in relation to the study species and design, as well as to the tissue of origin. The following are our suggestions for the identification of ILC3 by flow cytometry.

### 9.1 Mice: CD3^−^RORγt^+^

RORγt is the most reliable marker for ILC3 in mice. It is enough to use CD3 as the lineage marker in combination with RORγt in most of the studies. Additional lineage markers seem redundant as long as samples are not obtained from the lungs or spleen when neutrophil or myeloid/DC exclusion is needed. CD127 is required as an additional positive marker only if ILC1 and ILC2 are identified in parallel. CD90 should not be used as an exclusive ILC3 marker.

### 9.2 Humans: CD3^−^CD127^+^CD117^+^CD294^−^

CD127 in combination with T-cell-specific lineage markers is a proper way to distinguish human ILC from NK cells. CD294 is an ILC2-specific marker and is a good marker to discriminate between ILC2 and ILC3. As for the distinction of ILC1, CD117 is expressed on ILC2 and ILC3, but not on ILC1. The addition of CD161 is not necessary. It can be used instead of CD127, but in that case, additional lineage markers for myeloid cells are required. RORγt cannot be used for distinguishing ILC3 from other innate lymphocytes in humans.

### 9.3 General

There is no need to use CD45 when analyzing cells isolated from the blood, lymph nodes, spleen, or tonsils, but it is necessary to use it in quantitative analysis of tissue-residing ILC3.

In a number of studies analyzed in this review, we observe a superfluous use of lineage cocktail markers. Although unnecessary, such overapplication of markers does not jeopardize proper ILC3 identification. On the other hand, there are studies in which ILC3 is identified by an insufficient number of markers or inappropriate marker combinations. This raises the question of the accuracy of the results and the conclusions of such studies. We hope that this paper will help researchers interested in ILC3-related studies to tailor their research design to the best of their specific needs.

## Author contributions

ĐMil: Conceptualization, Funding acquisition, Writing – original draft, Writing – review & editing. IK: Visualization, Writing – original draft, Writing – review & editing. SS: Visualization, Writing – original draft. BJ: Writing – original draft, Writing – review & editing. DMic: Writing – original draft, Writing – review & editing. IS: Conceptualization, Writing – original draft, Writing – review & editing.
